# Sleep Disturbances and Their Associations with Child and Family Quality of Life in School-Aged Children Born Before 32 Weeks of Gestation: A Cross-Sectional Comparative Study

**DOI:** 10.3390/children13091222

**Published:** 2026-09-10

**Authors:** Ercan Yılmaz, Nezihe Koker Ozer, Hatice Turgut, Erdem Topal, Ramazan Özdemir, Recep Günakın, Mehmet Aslan

**Affiliations:** 1 Department of Pediatric Allergy and Immunology, Faculty of Medicine, Inonu University, Malatya 44210, Turkey; 2 Department of Neonatology, Faculty of Medicine, Inonu University, Malatya 44280, Turkey; 3 Department of Pediatric, Faculty of Medicine, Inonu University, Malatya 44280, Turkey

**Keywords:** PAQ-C, PedsQL, preterm birth, SDSC, sleep disturbances

## Abstract

**Highlights:**

**What are the main findings?**

- Children born before 32 weeks had more sleep disturbances than term-born peers.
- Difficulties initiating/maintaining sleep and sleep–wake transition disorders were more frequent.
- Sleep disturbances independently predicted poorer parental quality of life.

**What are the implications of the main findings?**

- Routine sleep screening should be incorporated into long-term follow-up of very preterm children.
- Family-centred interventions addressing sleep may improve parental well-being.

**Abstract:**

**Aim:** To compare sleep disturbances, quality of life, and physical activity levels between school-aged children born before 32 weeks of gestation and healthy term-born peers, and to examine the associations of sleep disturbances with physical activity and quality of life in both children and their parents. **Methods:** This cross-sectional comparative study included 55 children aged 7–8 years who were born before 32 weeks of gestation and 55 healthy term-born controls. Controls were frequency matched to the preterm group by age and sex to ensure comparable distributions of these key demographic characteristics. Sleep disturbances were assessed using the Sleep Disturbance Scale for Children (SDSC), children’s quality of life using the Pediatric Quality of Life Inventory (PedsQL), parental quality of life using the PedsQL Family Impact Module, and physical activity using the Physical Activity Questionnaire for Older Children (PAQ-C). **Results:** The total SDSC score was significantly higher in preterm children than in the control group (*p* = 0.044). Significant differences were observed particularly in the subscales assessing difficulties initiating and maintaining sleep (*p* = 0.007) and sleep–wake transition disorders (*p* = 0.019). Regarding children’s quality of life, only the emotional functioning subscale was significantly poorer in the preterm group (*p* = 0.002). For parental quality of life, the emotional functioning (*p* = 0.01) and daily activities (*p* = 0.04) subscales were significantly impaired, whereas physical activity levels were comparable between the groups. No significant associations were found between sleep disturbance and either children’s quality of life or physical activity in the preterm group. In multivariable analysis, children’s quality of life and total sleep disturbance score were identified as independent predictors of parental quality of life. **Conclusions:** School-aged children born before 32 weeks of gestation experience a greater burden of sleep problems, particularly disturbances involving sleep initiation and maintenance and the transition between sleep and wakefulness. Sleep disturbances were independently associated with poorer parental quality of life, regardless of children’s quality of life. These results emphasize the need to incorporate regular evaluation of sleep and a family-centered perspective into the long-term care of children born preterm.

## 1. Introduction

Preterm birth continues to represent a major contributor to childhood morbidity and mortality globally. However, improvements in perinatal and neonatal care have substantially enhanced survival, especially among infants delivered before 32 weeks of gestation. However, this increase in survival has led to long-term health outcomes taking on greater importance. It has been shown that in very preterm infants, numerous adverse outcomes—such as chronic respiratory diseases, neurodevelopmental disorders, learning difficulties, behavioral problems and a reduced health-related quality of life—can persist into school-age and adolescence. For this reason, the follow-up of preterm infants is now planned to cover not only the first years of life but also functional and psychosocial outcomes during the school-age years [1,2,3].

Sleep is one of the essential physiological processes during childhood for physical growth, brain development, cognitive performance, emotional regulation and immune system function. Sleep disorders in school-aged children are associated with attention deficits, reduced academic achievement, behavioral problems, emotional difficulties and a decline in quality of life [4,5]. In preterm infants, it is thought that sleep patterns may be affected in the long term due to the immaturity of the central nervous system, prolonged stays in the neonatal intensive care unit, chronic lung disease and early-life environmental stressors [6].

Recent studies have shown that sleep disorders are more common in children born prematurely than in their term-born peers. Systematic reviews have reported that these children experience lower sleep quality, more frequent night-time awakenings, shorter sleep duration and a higher incidence of sleep-related respiratory disorders [6]. Consistent with these observations, studies employing the Sleep Disturbance Scale for Children (SDSC) have reported particularly prominent problems with sleep initiation and maintenance, together with disturbances in the sleep–wake transition [7]. However, the majority of existing studies have merely characterized sleep characteristics; they have not assessed the combined effects of these disorders on the child’s daily life and the family.

Health-related quality of life has gained increasing relevance as an indicator of long-term outcomes following preterm birth. This multidimensional construct reflects several aspects of a child’s well-being, including physical, emotional, social, and academic functioning. Previous cohort studies and systematic reviews have shown that quality of life in very preterm infants is lower than that of their healthy peers, particularly in psychosocial domains [3,8]. However, quality of life is not solely a consequence of preterm birth; it is influenced by numerous factors such as sleep patterns, chronic illnesses, neurodevelopmental conditions and family support. The relationship between these variables, however, has not yet been fully elucidated.

The effects of preterm birth are not limited to the child alone. The long-term health monitoring of these children places a significant psychological, social and economic burden on parents. Studies conducted in recent years have shown that anxiety, depression, the burden of care and a decline in quality of life among parents of preterm children can persist for years [9,10,11]. Recently published systematic reviews and meta-analyses have also demonstrated that parents’ quality of life is significantly affected, particularly in emotional domains [10]. For this reason, it is recommended that long-term follow-ups of preterm infants should assess not only child-centered but also family-centered outcomes.

Factors determining parents’ quality of life include their child’s chronic health problems, neurodevelopmental issues and behavioral difficulties [11,12]. However, the number of studies examining the impact of sleep disorders in children on parents’ quality of life is quite limited. Yet sleep disorders can directly alter family life by affecting a child’s daytime functioning and emotional state, whilst also representing an intervenable risk factor.

Physical activity is also one of the key determinants of child health, and it is known that there is a reciprocal relationship between sleep and quality of life [13,14]. However, there are very few studies that jointly assess physical activity, sleep disorders, child quality of life and family quality of life in school-aged children born very preterm. Furthermore, there is a lack of information regarding the factors that independently determine parental quality of life.

The present study examined differences in sleep disturbances, physical activity, and child and family quality of life between school-aged children born before 32 weeks of gestation and healthy term-born controls. Relationships among these outcomes were also explored, together with the independent factors related to parental quality of life.

## 2. Materials and Methods

### 2.1. Study Design and Participants

This cross-sectional comparative study was conducted at the Pediatric Allergy Outpatient Clinic, Department of Pediatric Allergy and Immunology, Inonu University Faculty of Medicine, a tertiary university hospital. Prior to the clinical recruitment period, the neonatal intensive care unit records and hospital electronic database were reviewed to identify potentially eligible children born before 32 weeks of gestation who had received neonatal care at Turgut Ozal Medical Center. Thus, the potentially eligible preterm cohort had been identified from existing medical records before participant recruitment commenced.

Following approval by the institutional ethics committee, the recruitment and clinical assessment phase was conducted between 1 July and 15 July 2026. During this period, the families of the previously identified potentially eligible children were contacted by telephone, informed about the study, and invited to attend the outpatient clinic. Children whose families agreed to participate underwent clinical assessment, and the study questionnaires were completed during the same outpatient visit.

Eligible participants were children aged 7–8 years who had been born before 32 weeks of gestation. A total of 185 potentially eligible children were identified from the neonatal intensive care unit records. Of these, 59 children whose families were successfully contacted and agreed to participate were invited to attend the outpatient clinic for further evaluation. Of these, 4 children were excluded because their families declined to provide informed consent. A total of 55 children fulfilling the eligibility criteria were ultimately included in the analysis. Healthy children born at term (≥37 weeks of gestation) and of a comparable age served as controls; they were recruited during outpatient visits for routine child health follow-up and had no known chronic medical conditions. Frequency matching based on age and sex was applied to the control group to achieve demographic distributions comparable to those of the preterm group. Frequency matching was preferred because matching was intended specifically to control for age and sex rather than to balance a broader set of covariates, and because the relatively small sample size could limit the robustness and efficiency of propensity score matching. For the preterm group, the inclusion criteria were gestational age < 32 weeks, age 7–8 years at the time of assessment and written informed parental consent. Children with congenital heart disease, cystic fibrosis, primary immunodeficiency, chromosomal or genetic syndromes, or incomplete questionnaire data were excluded.

### 2.2. Clinical Assessment

Perinatal and neonatal data for preterm infants were obtained retrospectively from hospital records.

Variables assessed included gestational age, birth weight, small-for-gestational-age status, bronchopulmonary dysplasia, neonatal sepsis, patent ductus arteriosus, intraventricular hemorrhage, apnea of prematurity, mechanical ventilation and its duration, CPAP therapy and its duration, duration of oxygen therapy, length of NICU stay, surfactant administration, and breastfeeding for more than six months. In addition, the children’s current history of allergic diseases (asthma, allergic rhinitis and eczema), family history of atopy, and neurodevelopmental morbidities such as motor development problems, visual impairment, hearing loss and special educational needs were recorded. Height and weight were obtained using standardized measurement procedures, and the corresponding z-scores were derived from national reference values specific to the child’s age and sex [15].

All evaluations were completed during a single outpatient visit at the Pediatric Allergy and Immunology Outpatient Clinic to ensure consistency in data collection.

### 2.3. Assessment of Sleep Disturbances

Sleep disturbances were assessed using the parent-completed Sleep Disturbance Scale for Children (SDSC) [16]. The Turkish version of the SDSC, which has been validated and shown to be reliable, was used in this study [17]. The SDSC is a 26-item questionnaire that evaluates children’s sleep characteristics over the preceding six weeks and is widely used to assess pediatric sleep disturbances. The instrument is organized into six domains: Disorders of Initiating and Maintaining Sleep, Sleep Breathing Disorders, Disorders of Arousal, Sleep–Wake Transition Disorders, Disorders of Excessive Somnolence, and Sleep Hyperhidrosis. Higher total SDSC scores indicate greater sleep disturbance.

### 2.4. Assessment of Quality of Life and Physical Activity

Children’s health-related quality of life was evaluated using the Pediatric Quality of Life Inventory Version 4.0 Generic Core Scale (PedsQL 4.0), which was completed by their parents. The study utilized the parent form of the PedsQL 4.0, for which validity and reliability in Turkish have been demonstrated. The scale consists of a total of 23 items assessing physical, emotional, social and school functioning [18,19].

Parental quality of life was assessed using the PedsQL Family Impact Module. The study utilized the parental form of the scale, for which validity and reliability in Turkish have been demonstrated. The scale consists of 36 items covering multiple aspects of parental and family functioning, including physical, emotional, social, and cognitive functioning, as well as communication, anxiety, daily activities, and family relationships [20,21]. For both scales, in accordance with the scoring system used in the study, higher scores indicate a poorer quality of life.

Physical activity levels in the participating children were evaluated using the Physical Activity Questionnaire for Older Children (PAQ-C). The validated and reliable Turkish version of the PAQ-C was used in this study. This nine-item self-report instrument evaluates moderate-to-vigorous physical activity among school-aged children during the preceding seven days [22,23]. All questionnaires were completed by parents during the same outpatient clinic visit.

### 2.5. Statistical Analysis

Statistical analyses were performed using IBM SPSS Statistics for Windows, Version 25.0 (IBM Corp., Armonk, NY, USA). Continuous data were first evaluated for distributional characteristics using the Shapiro–Wilk test and visual examination of the observed distributions. Variables meeting the assumption of normality are summarized as mean ± standard deviation (SD), while those showing a non-normal distribution are reported as median with interquartile range (IQR). Categorical data are summarized as frequencies and percentages. For comparisons between the study groups, continuous variables were analyzed with an independent-samples *t*-test when the normality assumption was satisfied and with the Mann–Whitney U test otherwise. Differences in categorical variables were evaluated using either the chi-square test or Fisher’s exact test, depending on the distribution of the data. Comparisons of individual questionnaire subscales were considered exploratory, and no formal adjustment for multiple comparisons was applied; therefore, *p* values for these analyses should be interpreted cautiously. Because the variables examined in the correlation analyses did not uniformly satisfy the assumptions for Pearson’s correlation, their relationships were assessed using Spearman’s rank correlation coefficients. Specifically, correlations among sleep disturbances, physical activity, and child quality of life were examined using this non-parametric approach. In addition, a multivariable linear regression model was fitted to the full study sample (n = 110; 55 children born preterm and 55 term-born controls) to determine which factors were independently associated with parental quality of life. The model included prematurity status (binary variable), total Sleep Disturbance Scale for Children (SDSC) score, child quality-of-life score, and physical activity score as independent variables. The findings from the regression analyses are presented as standardized β estimates, unstandardized coefficients (B), their corresponding 95% confidence intervals (CIs), and *p* values. Statistical significance was defined as a two-sided *p* value < 0.05 for all analyses.

## 3. Results

### 3.1. Demographic, Anthropometric, Perinatal, and Neonatal Characteristics

A total of 110 children participated in the study, comprising 55 children born preterm and 55 healthy term-born controls. Both groups had a median age of 7 years, with no statistically significant between-group differences in either age or sex distribution. Children born preterm had significantly lower height z-scores (−0.34 ± 1.11 vs. 0.40 ± 0.86, *p* < 0.001) and weight z-scores (−0.47 ± 1.49 vs. 0.11 ± 1.03, *p* = 0.019) than healthy controls. Breastfeeding for more than 6 months was also less frequent in the preterm group (45.5% vs. 90.9%, *p* < 0.001). No significant between-group differences were observed in consanguinity, tobacco smoke exposure, personal history of asthma, allergic rhinitis or eczema, or family history of these atopic conditions (all *p* > 0.05) (Table 1).

In the preterm group, gestational age at birth had a median value of 30 weeks (IQR, 5), while the corresponding median birth weight was 1.2 kg (IQR, 0.75). Thirteen children (23.6%) were small for gestational age, 53 (96.4%) were delivered by cesarean section, and 24 (43.6%) were from multiple pregnancies. Regarding neonatal morbidities, 33 (60%) had neonatal sepsis, 19 (34.5%) bronchopulmonary dysplasia, 13 (23.6%) patent ductus arteriosus, 12 (21.8%) intraventricular hemorrhage, and 22 (40.0%) apnea of prematurity. Twenty-two children (40.0%) required mechanical ventilation, while 50 (90.9%) received continuous positive airway pressure support. The median duration of oxygen therapy was 12 days (IQR, 17), and the median length of NICU stay was 50 days (IQR, 46). Surfactant was administered to 11 children (20.0%). The detailed perinatal and neonatal characteristics of the preterm group are presented in Table 2.

To assess the potential for selection bias, the available neonatal and demographic characteristics of children included in the final analysis (n = 55) were compared with those of children identified from the neonatal records but not included in the study (n = 130). The two groups were comparable with respect to sex, gestational age, birth weight, small-for-gestational-age status, neonatal sepsis, necrotizing enterocolitis, patent ductus arteriosus, intraventricular hemorrhage, apnea of prematurity, bronchopulmonary dysplasia, need for and duration of mechanical ventilation, and length of neonatal intensive care unit stay, with no statistically significant differences identified for any of these variables (all *p* > 0.05). The results of this comparison are presented in Supplementary Table S1.

### 3.2. Sleep Disturbances, Quality of Life, and Physical Activity

Total SDSC scores were significantly higher in the preterm group than in controls [42 (13) vs. 35.5 (10.75), *p* = 0.044]. Among the SDSC subscales, Disorders of Initiating and Maintaining Sleep [13 (7) vs. 10 (6), *p* = 0.007] and Sleep–Wake Transition Disorders [9 (5) vs. 7 (3), *p* = 0.019] were significantly higher in children born preterm, whereas no significant between-group differences were observed for Sleep Breathing Disorders, Disorders of Arousal, Disorders of Excessive Somnolence, or Sleep Hyperhidrosis (all *p* > 0.05).

The preterm group had a higher median total Child PedsQL score than the control group [28 (20) vs. 19 (22)], although this between-group difference did not reach statistical significance (*p* = 0.085). Among the Child PedsQL domains, only emotional functioning was significantly worse in children born preterm [9 (7) vs. 4 (8), *p* = 0.002], while physical, social, and school functioning did not differ between the groups. Similarly, the median Family PedsQL total score was higher in parents of preterm children than in parents of healthy controls [49 (45) vs. 36 (48)], although the difference was not statistically significant (*p* = 0.11). Emotional functioning [6 (9) vs. 3 (6), *p* = 0.010] and daily activities [6 (5) vs. 4 (6), *p* = 0.04] were significantly more impaired in the preterm group, whereas physical functioning, social functioning, cognitive functioning, communication, worry, and family relationships were comparable between groups.

Children born preterm had lower physical activity scores than healthy controls [33 (8.2) vs. 36 (15)], although the difference between the groups did not reach statistical significance (*p* = 0.084). Comparisons of sleep disturbances, child and family quality of life, and physical activity between the two groups are presented in Table 3.

### 3.3. Correlation Analysis

Spearman rank correlation analyses performed in the preterm group demonstrated no significant associations between total sleep disturbance scores and child quality-of-life scores (*r* = 0.019, *p* = 0.892), between total sleep disturbance scores and physical activity scores (*r* = −0.004, *p* = 0.978), or between physical activity scores and child quality-of-life scores (*r* = −0.215, *p* = 0.116).

### 3.4. Multivariable Linear Regression Analysis

To determine the independent factors related to parental quality-of-life scores, a multivariable linear regression model was constructed using data from the full study sample of 110 participants, including 55 children born preterm and 55 term-born controls. The overall regression model was statistically significant (F = 20.177, *p* < 0.001), explaining 43.7% of the variance in parental quality-of-life scores (R^2^ = 0.437; adjusted R^2^ = 0.415). Child quality-of-life score was the strongest independent predictor of parental quality of life (standardized β = 0.577, B = 1.136, 95% CI 0.839–1.432; *p* < 0.001), followed by total sleep disturbance score (standardized β = 0.243, B = 0.647, 95% CI 0.248–1.045; *p* = 0.002). In contrast, physical activity score (standardized β = −0.025, *p* = 0.739) and prematurity status (standardized β = 0.016, *p* = 0.839) were not independently associated with parental quality-of-life scores. Table 4 summarizes the findings of the multivariable linear regression model used to determine the factors independently related to parental quality of life.

## 4. Discussion

This study examined differences in sleep disturbances, child and parental quality of life, and physical activity between school-aged children born before 32 weeks of gestation and healthy children born at term. The main findings of the study indicate that preterm children had higher total sleep disturbance scores, and that this difference was primarily attributable to “Disorders of Initiating and Maintaining Sleep” and “Sleep–Wake Transition Disorders”. In contrast, whilst only the emotional functioning sub-dimension of children’s quality of life was affected, a marked deterioration was observed in parents’ quality of life, particularly in emotional functioning and activities of daily living. In the multivariable analysis, child quality of life and sleep disturbance scores were independently associated with parental quality of life, whereas preterm birth was not independently associated with parental quality of life within the variables included in the model. However, this finding should not be interpreted as indicating an absence of an association between preterm birth and parental quality of life, as potentially relevant factors such as socioeconomic status, social support, and parental physical and mental health were not assessed. Furthermore, given the cross-sectional design of the study, the observed associations do not establish causal relationships.

The significantly elevated total SDSC score observed among children born preterm is in line with findings reported in previous studies. Systematic reviews indicate that preterm birth is associated with poorer sleep quality, more frequent night-time awakenings and a higher prevalence of sleep disorders during childhood [6,24]. The fact that, in our study, “Disorders of Initiating and Maintaining Sleep” and “Sleep–Wake Transition Disorders” subscales in particular were affected suggests that the underlying problem in these children is difficulty in falling asleep and maintaining sleep continuity. Previous research has likewise identified insomnia-like symptoms as one of the most frequently observed persistent sleep difficulties among children born preterm [24,25]. In contrast, the absence of significant between-group differences in sleep-related breathing disorders and excessive daytime sleepiness may reflect the limited representation of children with severe neurological impairment or serious chronic lung disease in our study population.

Although the effect of preterm birth on sleep has not been fully elucidated, it is thought that immature brain development, environmental stimuli in the neonatal intensive care unit, repeated painful procedures and neonatal complications may have a lasting impact on sleep architecture [6,26]. For this reason, the routine assessment of sleep in the follow-up of school-aged preterm children is important not only for supporting sleep health but also for promoting neurobehavioral development.

Regarding children’s quality of life, overall PedsQL scores were comparable between the groups; however, children born preterm had significantly lower scores in the emotional functioning domain. Whilst serious physical sequelae have decreased with advances in neonatal care, it is known that emotional and behavioral problems persist into school age [3,12]. Our findings also support this view. The fact that no significant differences were found in physical, social and school functioning may be related to the exclusion of children with severe neurodevelopmental disorders from the study, the limited sample size, and advances in current neonatal care practices. Furthermore, the fact that impairments in emotional functioning are observed alongside sleep disorders suggests that there may be a reciprocal interaction between these two conditions [5].

An important contribution of the present study is the evidence it provides regarding parental quality of life. Although emotional functioning and activities of daily living were more significantly affected in parents of preterm children, multivariate analysis revealed that a history of preterm birth was not an independent predictor. Instead, the child’s quality of life and sleep disturbances emerged as the strongest predictors of parental quality of life. This finding suggests that the quality of family life is more closely linked to the child’s current functional status than to the biological effects of preterm birth. Similarly, systematic reviews by Treyvaud et al. and Yip et al. reported that parents’ quality of life is significantly affected by the child’s ongoing health problems and psychosocial difficulties [9,10]. Our findings support the importance of a family-centered follow-up approach and suggest that interventions aimed at improving children’s sleep and quality of life may also indirectly enhance parents’ quality of life [8,11,27].

In this study, levels of physical activity did not differ between groups and were not among the independent predictors of parental quality of life. Whilst the literature reports that the exercise capacity of preterm infants may be reduced, the findings regarding daily physical activity levels are inconsistent [28,29]. Physical activity is shaped not only by an individual’s physiological capacity but also by a range of environmental influences, including the school setting, family support, and socio-economic circumstances. Furthermore, the fact that physical activity was assessed in our study using the self-reported PAQ-C scale and that objective measurement methods were not employed may have prevented us from identifying minor differences. However, our results indicate that, in explaining parental quality of life, sleep disturbances and the child’s emotional well-being play a more prominent role than physical activity [14].

Although no significant association was found between sleep disorders, children’s quality of life and physical activity within the preterm group in the correlation analysis, it is noteworthy that, in the regression analysis, children’s quality of life and sleep disorders independently explained parents’ quality of life. This suggests that relationships which cannot be demonstrated by univariate analyses may emerge in multivariate models. From a clinical perspective, long-term follow-up of preterm infants requires the assessment not only of medical morbidities but also of sleep health, emotional functioning and the family’s quality of life.

The strengths of the study include the combined assessment of sleep, children’s quality of life, parents’ quality of life and physical activity within the same cohort; the collection of detailed neonatal data from hospital records; and the use of validated scales in all assessments. Furthermore, the identification of factors independently influencing parental quality of life through multivariate regression analysis enhances the clinical value of the study. Several limitations of the present study should be considered. First, the cross-sectional nature of this single-center study restricts the broader applicability of the results and does not allow conclusions regarding causality. Second, the modest sample size may have reduced the statistical power to identify relatively small between-group differences or associations. Third, sleep and physical activity were assessed using questionnaires rather than objective measures. Fourth, potential confounders, including parental mental health, socioeconomic status, social support, and parental health, were not assessed. Fifth, the relatively low participation rate may have introduced selection bias. Although included and non-included children did not differ in the available demographic, perinatal, and neonatal characteristics, differences in unmeasured factors cannot be excluded. Sixth, all questionnaires were completed by parents, which may have introduced common-rater bias and influenced the observed correlations. Child self-report measures were not available. Finally, the absence of a multiplicity correction across the numerous comparisons may have increased the likelihood of type I error. Consequently, results related to specific questionnaire subscales should be viewed as exploratory and interpreted with caution.

## 5. Conclusions

Among school-aged children born before 32 weeks of gestation, greater sleep disturbances and lower child quality of life were independently related to poorer parental quality of life. These findings highlight the importance of considering sleep health, emotional well-being, and family quality of life in the long-term follow-up of children born preterm. However, given the cross-sectional design and the potential influence of unmeasured socioeconomic, social, and parental health factors, these associations should not be interpreted as causal, and the independent contribution of preterm birth to parental quality of life requires further investigation in longitudinal studies.

## Figures and Tables

**Table 1:** Current demographic and anthropometric characteristics of children born preterm and healthy controls.

Category	Variable	Children Born Preterm n = 55	Healthy Control n = 55	*p*-Value
Demographics	Male sex, n (%)	23 (41.8)	26 (47.3)	0.565
	Age, years, median (interquartile range)	7 (0.5)	7 (1)	0.409
	Height z-score, mean ± SD	−0.34 ± 1.11	0.40 ± 0.86	<0.001
	Weight z-score, mean ± SD	−0.47 ± 1.49	0.11 ± 1.03	0.019
	Consanguineous marriage, n (%)	16 (29.1)	12 (21.8)	0.511
	Breastfeeding > 6 months, n (%)	25 (45.5)	50 (90.9)	<0.001
	Tobacco smoke exposure, n (%)	11(20)	13 (23.6)	0.817
Personal history of atopy	Asthma, n (%)	6 (10.9)	4 (7.3)	0.742
	Allergic rhinitis, n (%)	12 (21.8)	18 (32.7)	0.284
	History of eczema, n (%)	3 (5.5)	5 (9.1)	0.716
Family history of atopy	Asthma, n (%)	8 (14.5)	10 (18.2)	0.797
	Allergic rhinitis, n (%)	14 (25.5)	18 (32.7)	0.529
	History of eczema history, n (%)	8 (14.5)	5 (9.1)	0.555

**Table 2:** Perinatal and neonatal characteristics and current comorbidities of children born preterm.

Category	Variable	n (%)
Perinatal and neonatal characteristics	Gestational age at birth, weeks, median (IQR)	30 (5)
	Birth weight, kg, median (IQR)	1.2 (0.75)
	Small for gestational age	13 (23.6)
	Cesarean delivery	53 (96.4)
	Multiple pregnancy	24 (43.6)
	Neonatal sepsis	33 (60)
	Necrotizing enterocolitis	2 (3.6)
	Neonatal PDA	13 (23.6)
	Neonatal IVH	12 (21.8)
	Apnea of prematurity	22 (40)
	Bronchopulmonary dysplasia	19 (34.5)
	Mechanical ventilation	22 (40)
	Duration of mechanical ventilation, days, median (IQR)	0 (2)
	CPAP	50 (90.9)
	Duration of CPAP therapy, days, median (IQR)	8 (20)
	Duration of oxygen therapy, days, median (IQR)	12 (17)
	Length of NICU stay, days, median (IQR)	50 (46)
	Surfactant administration	11 (20)
Current comorbidities	Motor developmental problem	12 (21.8)
	Visual disorders	13 (23.6)
	Hearing impairment	3 (5.5)
	Special education report	6 (13)

CPAP: Continuous positive airway pressure, IQR: Interquartile range, IVH: Intraventricular hemorrhage, NICU: Neonatal intensive care unit, PDA: Patent ductus arteriosus.

**Table 3:** Comparison of child and family quality of life between preterm and healthy control children.

Variable	Children Born Preterm n = 55	Healthy Control n = 55	*p* Value
SDSC			
Total SDSC score, median (IQR)	42 (13)	35.5 (10.75)	0.044
Disorders of Initiating and Maintaining Sleep score, median (IQR)	13 (7)	10 (6)	0.007
Sleep Breathing Disorders score, median (IQR)	4 (2)	3(2)	0.65
Disorders of Arousal score, median (IQR)	3 (2)	3 (1)	0.57
Sleep–Wake Transition Disorders score, median (IQR)	9 (5)	7 (3)	0.019
Disorders of Excessive Somnolence score, median (IQR)	6 (4)	6 (3)	0.57
Sleep Hyperhidrosis score, median (IQR)	2 (2)	3 (2)	0.77
Child PedsQL			
Total score, median (IQR)	28 (20)	19 (22)	0.085
Physical functioning score, median (IQR)	10 (9)	8 (10)	0.24
Emotional functioning score, median (IQR)	9 (7)	4 (8)	0.002
Social functioning score, median (IQR)	2 (6)	3 (3.3)	0.92
School functioning score, median (IQR)	5 (6)	4 (8)	0.082
Family PedsQL			
Total score, median (IQR)	49 (45)	36 (48)	0.11
Physical functioning score, median (IQR)	7 (6)	7 (10)	0.58
Emotional functioning score, median (IQR)	6 (9)	3 (6)	0.01
Social functioning score, median (IQR)	4 (7)	3 (7)	0.17
Cognitive functioning score, median (IQR)	5 (7)	4 (10)	0.58
Communication score, median (IQR)	4 (6)	2 (4)	0.19
Worry score, median (IQR)	6 (8)	5 (10)	0.72
Daily activities score, median (IQR)	6 (5)	4 (6)	0.04
Family relationships score, median (IQR)	7 (11)	4 (9)	0.11
PAQ-C score	33 (8.2)	36 (15)	0.084

IQR: Interquartile range, PedsQL: Pediatric Quality of Life, PAQ-C: Physical Activity Questionnaire for Older Children, SDSC: Sleep Disturbance Scale for Children.

**Table 4:** Multivariable linear regression analysis of factors independently associated with family quality of life.

Variable	Standardized β	B (95% CI)	*p* Value
Child quality of life	0.577	1.136 (0.839–1.432)	<0.001
Sleep disturbance score	0.243	0.647 (0.248–1.045)	0.002
Physical activity score	−0.025	−0.092 (−0.639 to 0.455)	0.739
Prematurity	0.016	0.432 (−3.789 to 4.653)	0.839

## Data Availability

Data supporting the findings of this study can be obtained from the corresponding author upon reasonable request. The data are not publicly available due to legal or ethical reasons.

## Supplementary Materials

The following supporting information can be downloaded at: https://www.mdpi.com/article/10.3390/children13091222/s1. Table S1: Comparison of Perinatal and Neonatal Characteristics Between Preterm Children Included and Not Included in the Final Analysis.
