# Supplementary material for: Sleep Disturbances and Their Associations with Child and Family Quality of Life in School-Aged Children Born Before 32 Weeks of Gestation: A Cross-Sectional Comparative Study
Source: Children (Basel). 2026 Sep 10;13(9):1222. doi: 10.3390/children13091222 (PMC13605283; doi:10.3390/children13091222)
Supplement: Supplementary file 1 [file children-13-01222-s001.zip › children-4505908-supplementary.pdf]

Supplementary Table S1. Comparison of Perinatal and Neonatal Characteristics Between Preterm Children Included and Not Included in the Final Analysis

**Variables**	**Included in the final analysis (n = 55)**	***Not included in the final analysis (n = 130)**	**p value**
Male sex, n (%)	23 (41.8)	67 (51.5)	0.227
Gestational week, median (IQR)	30 (5)	30 (3)	0.878
Birth weight, kg, median (IQR)	1.2 (0.75)	1.28 (0.43)	0.644
Small for gestational age, n (%)	13 (23.6)	35 (26.9)	0.777
Neonatal sepsis, n (%)	33 (60)	62 (47.7)	0.126
Necrotizing enterocolitis, n (%)	2 (3.6)	12 (9.2)	0.237
Neonatal PDA, n (%)	13 (23.6)	25 (19.2)	0.632
Neonatal IVH, n (%)	12 (21.8)	20 (15.4)	0.398
Apnea of prematurity, n (%)	22 (40)	62 (47.7)	0.424
Bronchopulmonary dysplasia, n (%)	19 (34.5)	35 (26.9)	0.387
Mechanical ventilation, n (%)	22 (40)	48 (36.9)	0.819
Mechanical ventilation, days, median (IQR)	0 (2)	0 (2)	0.671
Length of NICU stay, days, median (IQR)	50 (46)	41 (30)	0.125

IQR: Interquartile range, IVH: Intraventricular hemorrhage, NICU: Neonatal intensive care unit, PDA: Patent ductus arteriosus

* The group not included in the final analysis comprised children who could not be contacted and those who were contacted but did not meet the study eligibility criteria or could not complete the required assessments.
